# Cadmium stress triggers significant metabolic reprogramming in *Enterococcus faecium* CX 2–6

**DOI:** 10.1016/j.csbj.2021.10.021

**Published:** 2021-10-18

**Authors:** Xin Cheng, Bowen Yang, Jinfang Zheng, Hongyu Wei, Xuehuan Feng, Yanbin Yin

**Affiliations:** a Institute of Applied Microbiology, Jiangxi Agricultural University, Nanchang 330045, China; b Nebraska Food for Health Center, Department of Food Science and Technology, University of Nebraska - Lincoln, Lincoln, NE 68588, USA

**Keywords:** Stress response, Heavy metal resistance, Prophage, Differential expression, Cadmium, ncRNA, sRNA

## Abstract

Heavy metal pollutions in the soils are increasingly threatening the global crop and food production. Using plant associated bacteria to remediate heavy metal contamination is a promising approach. We have isolated a cadmium (Cd) resistant *Enterococcus faecium* strain CX 2–6 from a heavy metal contaminated farmland. We have shown that: (i) CX 2–6 can tolerate cadmium (Cd) with a slower growth rate; (ii) The CX 2–6 complete genome is fully assembled using PacBio long reads; (iii) Differential expression analysis found 47% of CX 2–6 genes are significantly affected by Cd treatment and form three gene groups with distinct expression profiles; (iv) Differentially expressed genes (DEGs) form physically linked gene clusters in the CX 2–6 genome, and one of the gene clusters corresponds to a prophage that is unique to CX 2–6 and is strongly activated when Cd concentration is higher; (v) A majority of DEGs responding to Cd treatment are present in the core genome; and (vi) 55 noncoding RNA genes are identified and 49 of them are DEGs responding to cadmium stress. Our pan-genome analysis and comparative RNA-seq data analysis has significantly improved our understanding of the metabolic reprogramming of *E. faecium* CX 2–6 under Cd stress.

## Highlights

• A cadmium resistant strain of *Enterococcus faecium* CX 2–6 is sequenced.
• Differential expression analysis found 47% of CX 2–6 genes are significantly affected by Cd treatment.
• Differentially expressed genes (DEGs) form physically linked gene clusters in the CX 2–6 genome.
• A prophage is unique to CX 2–6 and is strongly activated by high Cd concentration.
• A majority of DEGs responding to Cd treatment are present in the core genome.

## Graphical abstract

**Figure:**
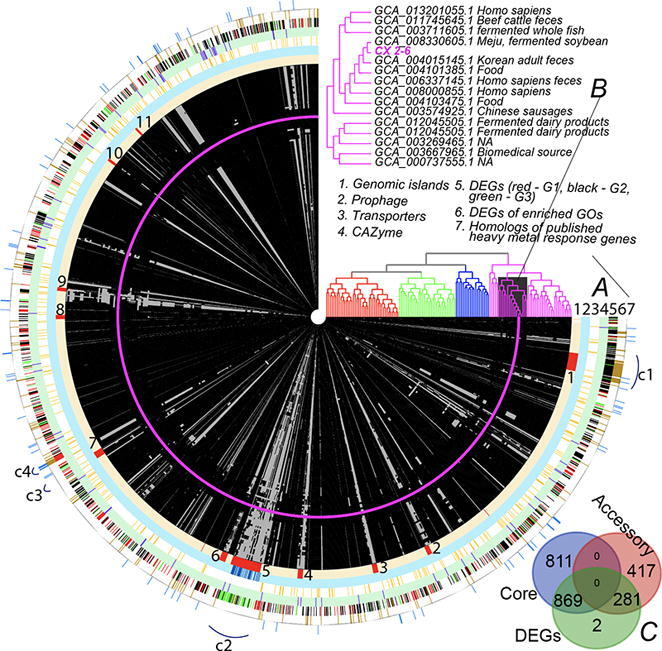

## 1 Introduction

Human activities and industrial developments have created tremendous stresses on the ecological environment. For example, as one type of abiotic stresses heavy metal pollutions in the soils are increasingly threatening the global crop and food production [1], [2], [3]. More importantly, consumption of foods and crops polluted by heavy metals can be a significant health risk to humans and animals [4]. For the remediation of soils contaminated with heavy metal pollutions, cheap, effective, and fast methods are in high demand and are currently being in extensive research [5], [6], [7].

Some plant associated bacteria can benefit plants in terms of heavy metal reduction and improved plant growth [7], [8]. For example, our recent research provided evidence that exopolysaccharides (EPS) from *Lactobacillus plantarum* can mediate the reduction of copper toxicity in rice seedlings, suggesting that EPS could be considered as a novel agent for effective heavy metal sequestration [9]. Others have also found that two strains of *Enterococcus faecium* commonly used in food industry can efficiently remove cadmium and lead from aqueous medium [10]. The importance of EPS has been also reported in gram-negative *Pantoea agglomerans* under the heavy metal stress*,* where EPS production is boosted together with a decrease in protein content and an increase in carbohydrate content [11].

Recently, different omics technologies have been increasingly used in the study of the metabolic response of various bacteria to heavy metals. For example, *Enterobacter* sp. DNB-S2 genome was sequenced to identify cadmium resistant genes [12]. Proteomics was used to study the probiotics *Lactococcus lactis* subsp. lactis for over-expressed proteins under cadmium stress [13]. By using comparative metabolomics [14] and comparative proteomics [15] approaches, the cadmium tolerance mechanism was investigated in different *Lactobacillus plantarum* strains. In addition to cadmium, stresses from other heavy metals have also been studied in various organisms using multi-omics technologies [16], [17], [18], [19], [20].

In this study, we have sequenced the genome of an *Enterococcus faecium* strain CX 2–6 isolated from a heavy metal contaminated farmland and performed a pan-genome and comparative transcriptome analysis. Our goal was to use pan-genome analysis and comparative RNA-seq data analysis to better understand the metabolic reprogramming of *E. faecium* CX 2–6 under the cadmium (Cd) stress. Compared to previous studies, our analyses have made a number of novel findings with respect to differentially expressed genes (DEGs), a novel prophage, and ncRNAs under cadmium stress. The main conclusion is that cadmium stress triggers significant metabolic reprogramming in *Enterococcus faecium* CX 2–6.

## 2 Materials and methods

### 2.1 Isolation and culture of *Enterococcus faecium* CX 2–6

Soil samples were collected from a rural rice paddy in Jiangxi Province, China and stored in acid sterilized plastic bags and kept at 4 °C before moved into −80 °C freezer in the laboratory. Samples were cultured under anaerobic conditions using an AnaeroPack-Anaero systems (Oxford, US) for 2–3 days at 34 °C.

Single colonies were streaked for purification on solid MRS plates. After that, all isolates were examined, and only Gram-positive and catalase- and oxidase-negative strains [21] were selected and stored at −80 °C in MRS broth with 20% glycerol. The morphology of isolated cells was observed under a light microscope and SEM (scanning electron microscope JSM-7001F, Japan Electronics Co., Ltd) (Fig. S1). The SEM was operated at 15–20 kV, with the magnifications of × 10,000, as described in Vinderola et al. [22].

All the isolates obtained were characterized by using 16S ribosomal RNA (rRNA) gene sequencing. The 16S rRNA sequences were compared to the NCBI nucleotide nr database to determine their identities. One of the Cd tolerant isolate was characterized as *Enterococcus faecium* CX 2–6 as its 16S rRNA (GenBank ID: MK788316) was 99.5% identical to other *E. faecium* 16S rRNAs or genomes. All isolates were stored at −80 °C.

To study the growth of CX 2–6, MRS medium was used to determine Cd tolerance at different concentrations. The pH was 7.2 with 1 M NaOH prior to sterilization; glucose solutions were sterilized by autoclaving at 117 °C for 20 min. Filter-sterilized CdCl2 solution was added to the medium after sterilization, and the final concentration was determined. Stock culture was transferred into MRS medium and incubated for 8 h at 34 °C. Then the culture was transferred to MRS medium and cultured at 37 °C for 48 h under anaerobic conditions. Optical density at 600 nm (OD600) was measured on a spectrophotometer (Model 721, Jingmi instrument, China) for cell growth determination.

To measure the exopolysaccharides (EPS) content of CX 2–6, sock culture was incubated in MRS medium for 8 h at 34 °C. Then the culture was transferred to EPS-producing medium (based on MRS medium, using sucrose as carbon source, which was more favored for the synthesis of EPS) and cultured at 37 °C for 48 h under anaerobic conditions. Extraction of EPS procedure has been described in our recent paper [9]. Briefly, 50 mL culture medium was centrifuged at 6000 rpm for 30 min. Supernatant was then collected and ethanol (analytical grade, 96%) was added. After 4 °C treatment for 24 h, the EPS precipitate was centrifuged for 30 min at 6000 rpm, washed with ethanol, and re-centrifuged. Precipitated EPS was dried at 80 °C for 2 h before weighing.

For DNA sequencing, the CX 2–6 isolate was inoculated and cultured in MRS medium at 34 °C for 8 h. Then the bacteria were collected by centrifugation at 6000× rpm for 10 min. The resulting pellet was washed with 1x PBS for two times, and immediately put into liquid nitrogen. The frozen bacteria were shipped with dry ice to the sequencing company.

For RNA sequencing, the CX 2–6 isolate was inoculated and cultured in MRS medium at 34 °C for 8 h. Then cadmium (CdCl_2_) was added into the medium. Three experimental conditions were used each with three replicates: cadmium at 0 (control), 1 mmol/L and 5 mmol/L, respectively. After medium were added, the cultures were kept at 34 °C for another 6 h. Then cultures were centrifuged at 6000× rpm for 10 min. After removing the supernatant, the bacteria pellet was washed with 1× PBS twice and frozen with liquid nitrogen immediately. The bacteria were shipped with dry ice to the sequencing company for RNA sequencing.

### 2.2 CX 2–6 genome sequencing, assembly, annotation

The DNA extraction, sequencing, and assembly were all performed by Guangzhou Gene Denovo Biotechnology Co. Ltd., China. SMRT sequencing was performed on the Pacific Biosciences RSII sequencer (PacBio, Menlo Park, CA) according to standard protocols (MagBead Standard Seq v2 loading, 1 × 180 min movie) using the P4-C2 chemistry. Continuous long reads were attained from three SMRT sequencing runs. Reads longer than 500 bp with a quality value over 0.75 were used for assembly.

The hierarchical genome-assembly process (HGAP) pipeline [23] was used to correct for sequencing errors in the reads. The resulting corrected, preassembled reads were used for de novo assembly using Celera Assembler with an overlap-layout-consensus (OLC) strategy [24]. The quality of the assembly was evaluated by the Quiver consensus algorithm [25] to determine the final genome sequence. Lastly, the two ends of the assembled sequence were aligned and trimmed to have the genome circularized. The fully assembled chromosome and plasmid sequences were submitted to GenBank (GenBank IDs will be available after the paper is published).

Protein coding genes were predicted using the GeneMarkS [26], a gene finding program for prokaryotic genomes. Repetitive elements were identified by RepeatMasker [27], rRNAs was predicted using rRNAmmer [28], and tRNAs were identified by tRNAscan [29].

Proteins were annotated by searching against various protein sequence and protein family databases, such as National Center for Biotechnology Information (NCBI) nonredundant protein (nr) database, UniProt/Swiss-Prot, Kyoto Encyclopedia of Genes and Genomes (KEGG), Gene Ontology (GO), Cluster of Orthologous Groups of proteins (COG), and protein family (Pfam) databases. In addition, prophages were predicted by PHASTER [30], genomic islands were predicted by IslandViewer4 [31], CAZymes were predicted by dbCAN [32], and transporters were annotated by searching against the TCDB database [33].

### 2.3 CX 2–6 RNA sequencing under Cd stress and differential expression analysis

The RNA extraction, sequencing, and expression quantification were all performed by Guangzhou Gene Denovo Biotechnology Co. Ltd., China. Total RNA was extracted by using the TRIzol-based method (Life Technologies, CA, USA). To construct libraries for sequencing, the rRNAs were depleted from 1 µg of total RNA using Illumina MRZB12424 Ribo-Zero rRNA Removal Kit (Bacteria) (Illumina, San Diego, CA, USA). Then, the first-strand cDNA was synthesized using ProtoScript Ⅱ Reverse Transcriptase (New England BioLabs, Ipswich, MA, USA) at 25 °C for 10 min; 42 °C for 15 min; 70 °C for 15 min. The second-strand cDNA was synthesized using NEBNext Second Strand Synthesis Reaction Buffer and dATP, dGTP, dCTP, dUTP mix (New England BioLabs, Ipswich, MA, USA) at 16 °C for 1 h. The second-strand cDNA was then degraded using the USER enzyme mix (New England BioLabs, Ipswich, MA, USA) at 37 °C for 15 min and the product was purified by Agencourt AMPure XP beads (Beckman Coulter, Brea, CA). Then the sequencing library was constructed using NEBNext® Poly(A) mRNA Magnetic Isolation Module (New England Biolabs, Ipswich, MA, USA). Sequencing was performed using the Illumina Novaseq 6000 platform with paired-end 150 base reads.

Raw data were filtered by the following standards: (1) removing reads with ≥10% unidentified nucleotides (N); (2) removing reads with >50% bases having Phred quality scores of ≤20; (3) removing reads aligned to the barcode adapter using FASTP (https://github.com/OpenGene/fastp). Quality trimmed reads were mapped to the reference genome using Bowtie2 [34] (version 2.2.8) allowing no mismatches. Reads mapped to rRNA were removed. Retained reads were used to calculate gene expression values by RSEM [35]. The cleaned RNA-seq reads were submitted to NCBI SRA (SRAs ID will be available after the paper is published).

The gene expression level was further normalized by using the fragments per kilobase of transcript per million (FPKM) mapped reads method to eliminate the influence of different gene lengths and amount of sequencing data on the calculation of gene expression. The edgeR [36] package was used to identify differentially expressed genes (DEGs) across samples with fold changes ≥2 and a false discovery rate-adjusted P (q value) <0.05. DEGs were then subjected to an enrichment analysis of GO function and KEGG pathways, and q values were corrected using <0.05 as threshold.

### 2.4 Noncoding RNA prediction and expression analysis

The prediction of ncRNA was conducted using Rockhopper [37] with the removal of sequences <50 bp. Candidate ncRNAs were then screened against the sRNAMap [38] and Rfam [39] database for annotation. Secondary structures of ncRNA were predicted by Vienna RNA packages [40] (version 2.3.5). The ncRNA expression level was normalized by using TPM values (transcripts per million). The edgeR package was used to identify differentially expressed ncRNAs across samples with fold changes ≥2 and a false discovery rate-adjusted P (q value) <0.05.

### 2.5 Pan-genome analysis

As of May 2020, the species *E. faecium* (NCBI Taxonomy ID: 1352) has 2081 genomes in the RefSeq database, among which 137 are fully assembled into complete genomes while others are in contig or scaffold draft genome status.

A pan-genome analysis was performed on the 137 complete genomes (Table S1) plus our CX 2–6 genome using anvi’o [41]. A gene family (or cluster) is defined in anvi’o as a group of genes from different genomes sharing significant sequence similarities among each other. By examining gene families consisting of members from different genomes, one can extract core genes (gene families with members from all the genomes), accessary genes (gene families with members from partial genomes), and ORFan genes [42], [43] (gene families only found in one genome).

## 3 Results

### 3.1 *E. faecium* CX 2–6 can tolerate cadmium (Cd) with a slower growth rate

**Fig. 1:**
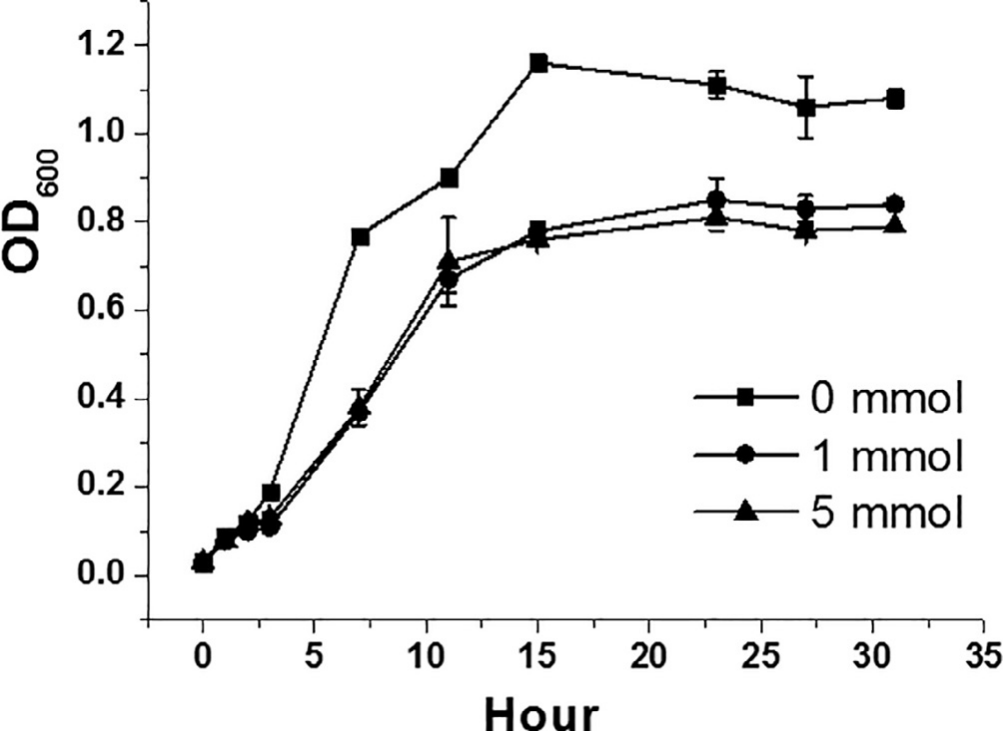
Growth curves of CX 2–6 under different Cd treatments. To understand how CX 2–6 responds to heavy metals, the bacteria were grown in three different media with cadmium (CdCl_2_) under a concentration at 0 (control), 1 (medium) mmol/L, and 5 (high) mmol/L, respectively. Each treatment had 3 replicates.

We have isolated multiple strains of bacteria from a rural farmland in Jiangxi Province of China, which is known to be very heavy metal contaminated. One of these strains was determined to be a new strain (named CX 2–6) of ***E. faecium*** based on a 16S rRNA (GenBank ID: MK788316) sequencing and comparison. From the growth curve (Fig. 1), we can observe that the bacterial growth is significantly decreased by Cd. The control group had the highest growth rate, while the Cd treated groups had much lower growth rates (not much difference between Cd1 and Cd5 though).

### 3.2 CX 2–6 complete genome was obtained by a PacBio long read sequencing and assembly

The CX 2–6 genome was then sequenced by PacBio long read sequencing technology with a depth 345X. In total 102,797 reads with a mean length = 9,142 bp were obtained and assembled into a complete chromosome (length = 2,559,204 bp) and a complete plasmid (length = 168,866 bp). In addition, we have also sequenced the RNA-seq transcriptomes of CX 2–6 with three conditions: control, cadmium (CdCl_2_) concentration at 1 mmol/L, and at 5 mmol/L. Each condition had three replicates and therefore in total nine RNA samples were sequenced with a sequencing depth ranging from 799 to 931X. After quality trimming, between 14,510,040 and 16,916,746 clean paired-end Illumina reads (2 × 150 bp) of the nine samples were mapped to the complete genome of CX 2–6 for expression analysis.

Gene prediction found 2475 protein coding genes in the main chromosome and 209 protein coding genes in the plasmid. Additionally, the main chromosome also encodes 67 tRNAs, six 16 s rRNAs, six 5 s rRNAs, and six 23 s rRNAs. Genomic island (GI) prediction found 11 GIs in the main chromosome and 4 GIs in the plasmids (Table S1). GIs are thought to be horizontally transferred and confer selective advantages to their host for environmental adaptation. CRISPRCasFinder [44] did not find any complete CRISPR-Cas systems in this genome. PHASTER found a prophage in the main chromosome (37,516 bp encoding 53 genes). In addition, 290 proteins were transporters according to a search against TCDB, and 74 proteins were carbohydrate active enzymes (CAZymes) by dbCAN2. In this paper, we will focus on genes and genetic elements (e.g., GIs) encoded in the main chromosome. We will study their gene expression changes under Cd stress, as well as their conservation among other sequenced genomes of *E. faecium* through a pan-genome analysis.

### 3.3 Differential expression analysis found 47% genes are significantly affected by Cd treatment and form three groups with distinct expression profiles

**Fig. 2:**
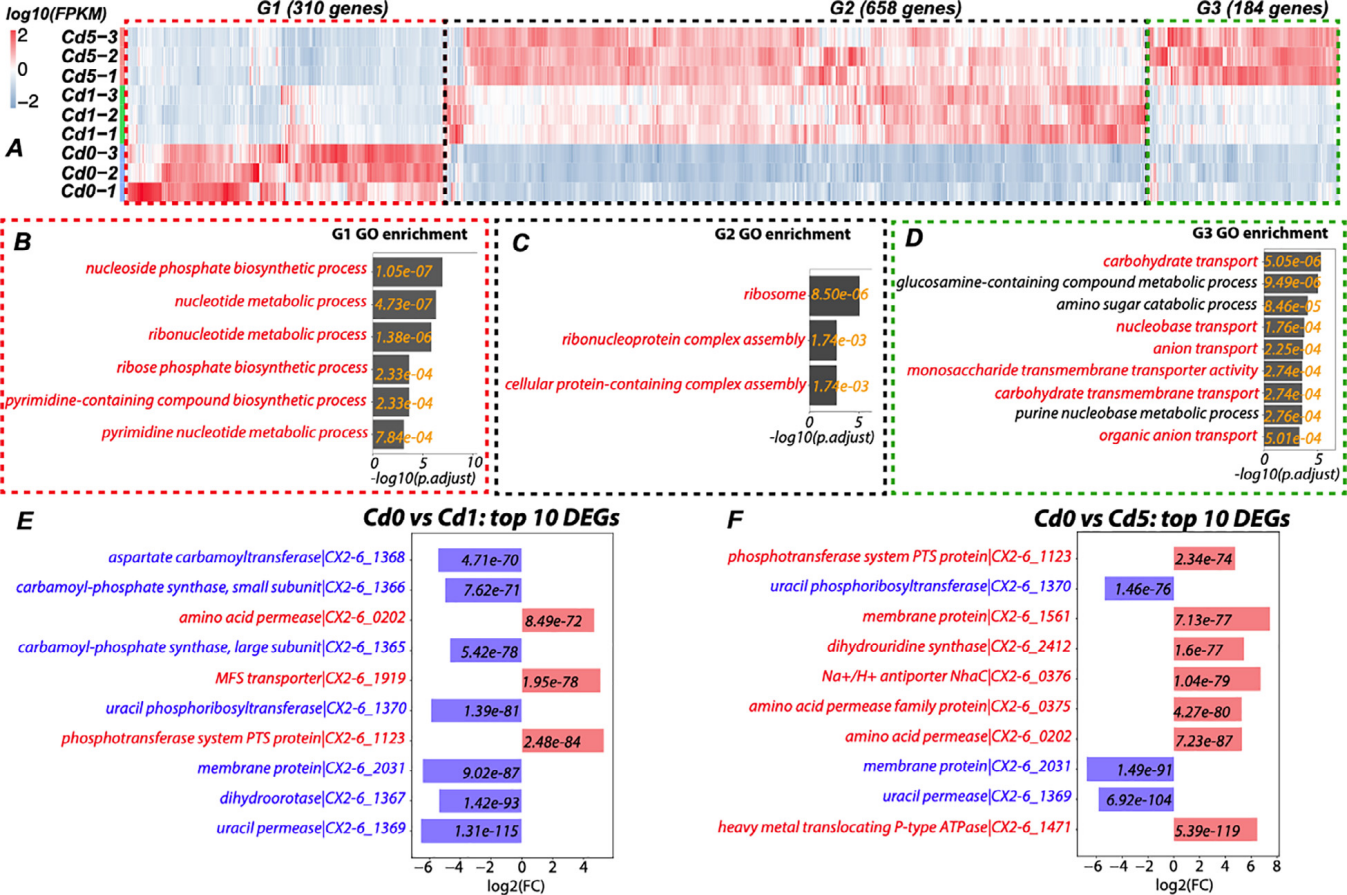
Differentially expressed genes (DEGs) in the CX 2–6 main chromosome. (A): 1152 DEGs are classified into G1, G2, and G3 groups according to distinct expression profiles; (B): Significantly (adjusted P-value <0.01) over-represented GO functional groups in G1 genes; (C): Significantly over-represented GO functional groups in G2 genes; and (D): Significantly over-represented GO functional groups in G3 genes. P-values were calculated by binominal tests and multiple comparisons were corrected to derive adjusted P-values; (E): The top 10 most significant DEGs (ranked by adjusted P-value) identified between Cd0 and Cd1 RNA-seq comparison; (F): The top 10 most significant DEGs identified between Cd0 and Cd5 RNA-seq comparison. Blue genes are down-regulated and form a gene cluster and encode enzymes for the pyrimidine metabolism. Red genes are up-regulated and encode transporters. (For interpretation of the references to color in this figure legend, the reader is referred to the web version of this article.)

Mapping the RNA-seq reads to the genome found that seven of the nine samples have a mapping rate >96% and the other two have a mapping rate >91%. On average, 92% of protein coding genes are expressed under the three conditions. Differential gene expression analysis found 891 significantly differentially expressed genes (DEGs) with a false discovery rate (FDR) < 0.05 and |log2FC(fold change)|>1, including 687 up-regulated DEGs and 204 down-regulated DEGs for the Cd0-vs-Cd1 comparison. Similarly, 1009 DEGs (FDR < 0.05 and |log2FC|>1) were found for Cd0-vs-Cd5, including 744 up-regulated DEGs and 265 down-regulated DEGs; and 203 DEGs (FDR < 0.05 and |log2FC| > 1) were found for Cd1-vs-Cd5, including 140 up-regulated DEGs and 63 down-regulated DEGs. In total, 1152 (47%) genes of the main chromosome are significant DEGs in at least one condition pairs. Clustering all these 1,152 DEGs and presenting them in a heatmap (Fig. 2A) clearly shows that they form three groups of genes based on shared expression profiles. Group 1 (G1) contains 310 genes down-regulated by Cd treatment, group 2 (G2) contains 658 genes up-regulated by Cd treatment at the low concentration (Cd1), while group 3 (G3) contains 184 genes highly up-regulated by Cd treatment at the high concentration (Cd5).

Gene ontology (GO) enrichment analysis was performed on the three groups of genes by using binomial tests. For the tests, the background includes all the 2475 genes, and the foreground includes G1, G2, or G3 genes, respectively. For example, for G1 genes, 310 significantly down-regulated with Cd were assigned to 43 GO terms. For each GO term we counted the number genes (denoted as n) in the foreground (from the 310 G1 genes) assigned to this GO term. We also counted the number of genes (denoted as N) in the background (from the 2475 total genes) assigned to this GO term. Using these four numbers (n, 310, N, and 2475), a binomial test P-value was computed for the GO term to evaluate how enriched this GO function is in the foreground compared to the background. After all the 43 GO terms were computed, a multi-testing correction was conducted to obtain the adjusted P-values, and GO terms with adjusted P-value <0.01 were shown in Fig. 2B. The G1 result indicates that the nucleotide metabolism-related GO terms are significantly enriched in G1 genes (Fig. 2B). This means that nucleotide metabolism is significantly repressed when CX 2–6 is treated with Cd, meaning DNA replication is inhibited, which is consistent with the observed slower bacterial growth (Fig. 1). In contrast, GO enrichment analysis on G2 genes found that ribosome related GO functions are significantly enriched in G2 (Fig. 2C), suggesting protein translation is increased once cells are exposed to low concentration of Cd. Interestingly, GO enrichment analysis on G3 genes (Fig. 2D) found that only when Cd concentration is higher, carbohydrate and anion transporting activities are increased. Our data also showed an increased exopolysaccharide production under Cd stress (Fig. S2). These might be because the highly stressed cells need carbohydrates for Cd sequestration, anions for Cd neutralization, and increased antiporter and ATPase activities to pump Cd out of the bacterial cells.

Fig. 2E and F show the top 10 DEGs ranked by P-values in comparisons of RNA-seq of Cd0-vs-Cd1, and Cd0-vs-Cd5, respectively. Interestingly, for the Cd0-vs-Cd1 comparison (Fig. 2E), all the top down-regulated genes (blue color) are physically linked (CX2-6_1165 to CX2-6_1170), form a gene cluster in the genome and encode enzymes related to nucleotide metabolism, which agrees with the findings made in Fig. 2B. In contrast, the top up-regulated genes (red color) mostly encode transporters. For the Cd0-vs-Cd5 comparison (Fig. 2F), the top 10 DEGs include seven up-regulated genes (red color), all but one encoding transporters.

### 3.4 DEGs form physically linked gene clusters in the CX 2–6 genome

**Fig. 3:**
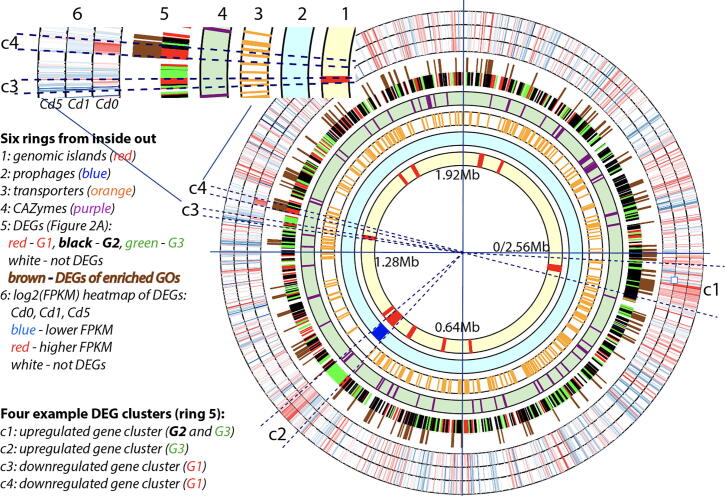
Circos plot of the CX 2–6 main chromosome with different rings to highlight various genes and genetic elements. The six rings were explained in the legend. The six rings were separated with white spaces. The 5th ring includes two layers. The 6th ring includes three layers. Four DEG clusters were identified by visual inspection and explained in the legend. Two clusters (c3 and c4) were shown in an enlarged view with the six rings labeled.

To further investigate DEG clustering in the genome, all the 1152 DEGs were mapped onto the circular chromosome. The GIs, prophage, transporters, CAZymes, and DEGs were located in the genome and displayed using a Circos plot (Fig. 3). The plot reveals a number of highly differentially expressed and physically linked gene clusters. Four of the most evident gene clusters are elaborated as examples.

The first DEG cluster (c1 in Fig. 3) is the largest (89 genes) and contains the GI-1 (36 genes, CX2-6_0054 - CX2-6_0089). GI-1 (Table S2) is largely the operon encoding ribosomal proteins to build the ribosomal small and large subunits, which are known to be highly expressed. Indeed, this region in the genome has almost the highest expression level (more red color in the 6th ring of Fig. 3). This ribosomal protein operon was in fact falsely predicted as a GI by IslandViewer4, as its algorithm looks for atypical nucleotide composition, a feature that both horizontally transfer genes and highly expressed genes tend to have. From the 5th ring of Fig. 3, it is also clear that surrounding this GI-1, there are also other significantly up-regulated DEGs (black and green colors in the 5th ring), which together form an almost consecutive (almost no red and white genes) up-regulated DEG cluster. These non-GI-1 genes are mostly encoding membrane transport for environmental information processing or metabolic enzymes for carbohydrate metabolism according to KEGG pathway annotation (Table S2). For example, 17 transporters are encoded by these non-GI-1 genes, most of which are significantly up-regulated DEGs.

The second DEG cluster (c2 in Fig. 3, 53 genes, CX2-6_0917-CX2-6_0969, Table S3) aligns perfectly with the predicted prophage (the 2nd ring), which also corresponds to GI-5, the 5th of the 11 predicted GIs (the 1st ring). Interestingly, most genes in this prophage or GI-5 are significantly up-regulated by Cd treatment at the high concentration (green in the 5th ring of Fig. 3). The left end (5′ end) of the prophage seems to have very high expression level (red in the 6th ring) even when there is no Cd in presence. When CX 2–6 is treated with Cd particularly at a higher concentration, the prophage is expressed at a very high level. None of the genes in this prophage encode transporter proteins, but one CAZyme gene was found to encode a GH23 (glycoside hydrolase family 23) enzyme, which is a peptidoglycan lyase to break bacterial cell walls.

The third DEG cluster (c3 in Fig. 3, 14 genes, CX2-6_1328 - CX2-6_1341) is much smaller but overlaps with GI-7. It is one of the very few down-regulated gene clusters under Cd stress (red colors in the 5th ring). In fact, not all the genes in c3 are down-regulated and most of them have very low baseline expression (Table S4). In contrast the most highly expressed genes are all significantly repressed with Cd treatment. These repressed genes include genes encoding the phosphoenolpyruvate (PEP)-dependent phosphotransferase system that is a well characterized for simple carbohydrate (e.g., monosaccharide or disaccharide) update/transport.

The fourth DEG cluster (c4 in Fig. 3, CX2-6_1362 - CX2-6_1370) contains 9 genes, which form an operon encoding enzymes for pyrimidine metabolism (Table S5). This DEG cluster contains most of the down-regulated DEGs shown in Fig. 2E and F. In agreement with that, the 6th ring of Fig. 3 shows that these genes are expressed at a very high level in Cd0, but at a much lower level at Cd1 and Cd5.

### 3.5 A majority of DEGs responding to Cd treatment are found in the core genome

Pan-genome is an important concept in bacterial comparative genomics, consisting of all the genes encoded in multiple strains of the same species. A pan-genome analysis involves comparing the gene contents of all the genomes of a species to find genes that are present in all the genomes (core genes), genes that are unique to a single genome (ORFan genes), and genes present in some but not all genomes (variable or accessory genes).

**Fig. 4:**
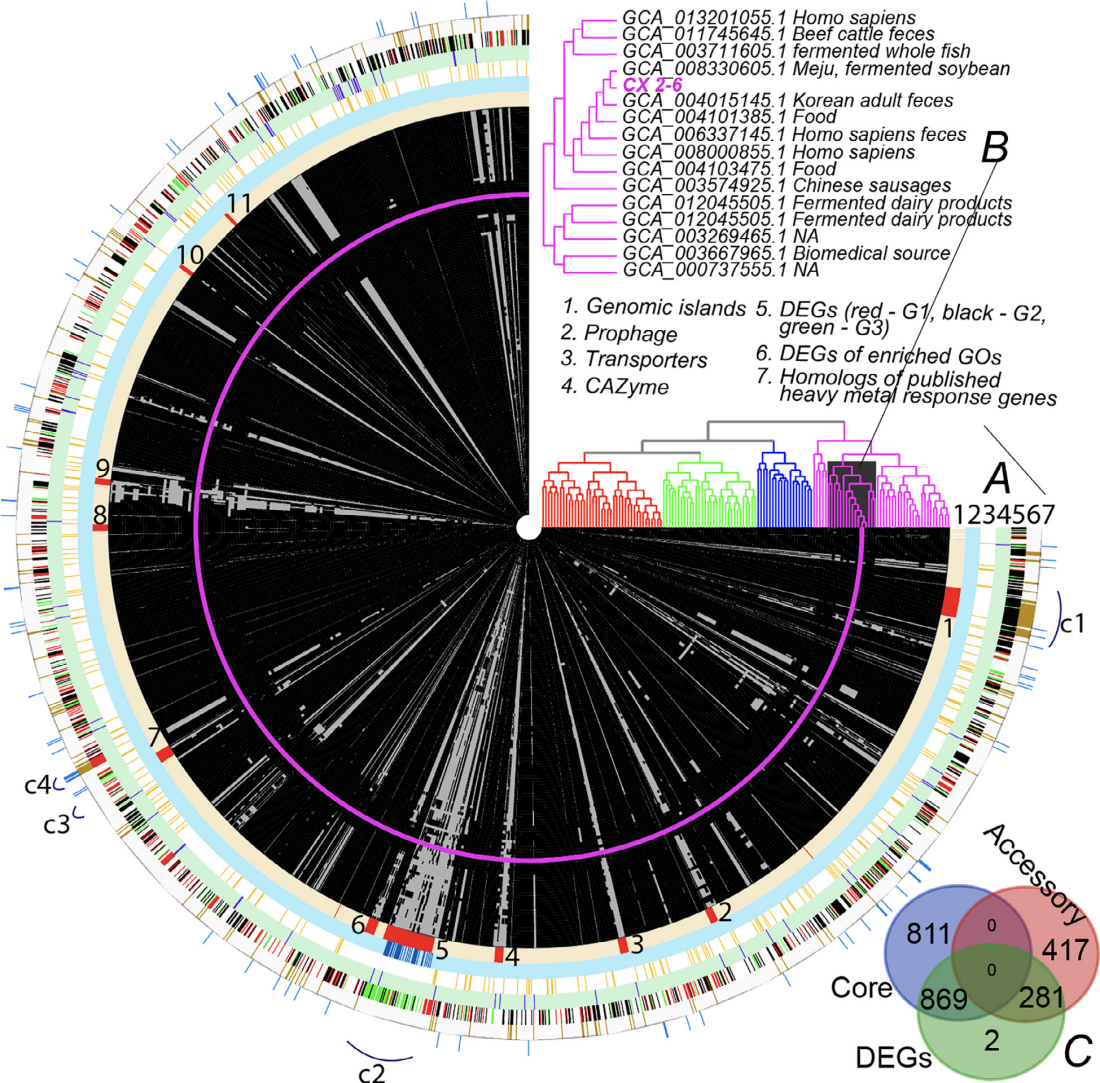
Anvi’o plot of the pan-genome of 138 E. faecium genomes. From the innermost, each circle represents a genome, and therefore there are in total 138 circles (each consists of gene families as tiny black boxes). Except for CX 2–6 (pink circle consisting tiny boxes [gene families]), in all other circles each tiny black box means the genome contains the gene family and grey box means the genome does not contains the gene family. The CX 2–6 circle is all pink, because only the genes families found in CX 2–6 are plotted here. Fig. S3 provides all genes families found in the 138 genomes including those absent in CX 2–6. The gene families are arranged according to their positions in the CX 2–6 genome. (A): Seven rings are used to show the interested genes and genetic elements, as what are shown in Fig. 3. (B): The gene presence/absence tree is shown, reflecting the evolutionary relationship among the 138 genomes. Each branch of the tree aligns with each genome circle. From this tree, four major groups of genomes are identified and marked with different branch colors. A sub-group of genomes (pink branches with black background) sharing the similar gene presence/absence pattern are visually identified. An enlarged tree view of this group is shown together with the GenBank Assembly ID and the isolation site/location information (extracted from the NCBI BioSample database). (C): Venn diagram of core genes, accessary genes and DEGs of CX 2–6. (For interpretation of the references to color in this figure legend, the reader is referred to the web version of this article.)

Fig. 4A presents 7 rings with gene families containing the GIs, prophage, transporters, CAZymes, and DEGs identified in the 138 complete genomes (Table S1, Methods). All gene families are arranged in the order of their linear positions in the genome of CX 2–6, so that we can inspect the conservation (presence and absence) of each gene family and each physically linked gene cluster across the 138 ***E. faecium*** genomes. In addition, a genome phylogeny (Fig. 4B) is built based on the gene presence/absence information across all gene families in the 138 genomes.

In total 7166 gene families (shown in Fig. S3) are classified by anvi’o in the 138 genomes, and 2483 (shown in Fig. 4) gene families are found in CX 2–6. Among these 2483 gene families of CX 2–6, 1636 (66%) are core genes, 5 (0.2%) are ORFan genes, and the rest are accessary genes. Interestingly, we found that out of the 1152 DEGs of CX 2–6, 869 are core genes present in all the 138 genomes, and 281 are accessory genes conserved in ≥2 genomes (Fig. 4C), suggesting Cd treatment mainly affects more conserved genes.

The 138 genomes form four major groups (red, blue, green, and pink colors in the dendrogram of Fig. 4B). There is a sub-group of genomes (16 in total, pink color with black background and with an enlarged view on the top) that share a very similar gene family presence/absence profile as CX 2–6. These genomes are sequenced from ***E. faecium*** strains isolated from mostly fermented foods or animal feces and geologically from East Asia (Table S1). This suggests that CX 2–6, while isolated from soil of a farmland, may be originated from a food production chain in East Asia.

Interestingly, most of the CX 2–6 GIs (e.g., GI-2, 3, 4, 5, 6, 9, 10) are narrowly distributed in the pink sub-group of genomes closely related to CX 2–6 (Fig. 4A, ring 1). However, some of the GIs (e.g., GI-3, 4, 5, 9) seem to have most of their genes uniquely present in CX 2–6. This has been verified by a search against all 2,371 *E. faecium* (complete and draft) genomes available in RefSeq database, which confirmed that only GI-1 is conserved while others are not. As for the four DEG clusters, c1 (GI-1) is the most conserved, with most of its genes present in all the genomes; c2 (GI-5 and prophage) is the least conserved and has most of its genes absent in other genomes; c3 and c4 are intermediate in sequence conservation but seem very conserved in the sub-group of genomes closely related to CX 2–6.

The five ORFans (unique genes in CX 2–6 not present in the other 137 genomes) are all located in GIs. CX2-6_0837 and CX2-6_0840 are located in GI-4, while CX2-6_0927, CX2-6_0928, and CX2-6_0930 are located in GI-5 (prophage). Being in the GIs suggests that these ORFan genes might be horizontally transferred from other bacterial species than ***E. faecium***. Searching these genes in the RefSeq bacterial database actually found hits for all the five ORFans. The small number of top hits are mostly from other strains of ***E. faecium*** (e.g., those with draft genomes not included in our 137 complete genomes), and more hits are from other species of the *Lactobacillales* order, suggesting that gene transfers may have happened from other bacteria. Interestingly, two (CX2-6_0840 [glycosyltransferase family 2 protein of CAZymes] and CX2-6_0930 [phage protein]) of the five ORFans responded to the increase of Cd level (i.e., DEGs in Cd1-vs-Cd5 comparison).

In addition, we have collected 121 previously published Cd response genes from literature (Fig. 4A, ring 7, Table S6). All these genes are from *Lactobacillus plantarum* or *Lactococcus lactis* [13], [15], which belong to the same *Lactobacillales* order as ***E. faecium***. A BLASTP search found 95 (78.5%) of the 121 published Cd response proteins have 218 homologs in the CX 2–6 genome (E-value < 1e−10). Interestingly, 129 (59.2%) of the 218 CX 2–6 homologous genes are also DEGs in our study, and 174 (79.8%) of them are also core genes.

### 3.6 55 noncoding RNA genes are identified and 49 of them are DEGs responding to cadmium stress

In addition to protein-coding genes, we have also identified noncoding RNA (ncRNA) genes based on RNA-seq mapping result. Noncoding RNAs regulate the expression of target mRNAs through complementary base-pairings or binding to chaperone proteins [45]. In this study, we have focused on two major types of ncRNAs: anti-sense RNAs (asRNAs) that are on the opposite strand of mRNAs, and intergenic RNAs (igRNAs) that are located in the intergenic regions of mRNAs. To identify ncRNAs, Rockhopper [37] was used to map RNA-seq reads to the genome; reads mapped to the intergenic regions or the opposite strand of mRNAs were assembled into novel transcripts. From these novel transcripts, ncRNAs were identified as those that (i) are >50 bp long, (ii) do not have protein homology in the NR database, (iii) have stable step-loop secondary structures, and (iv) do not have overlap with other mRNAs at all (ig-sRNAs) or have full or partial overlap with other mRNAs on the opposite strand (asRNAs).

**Fig. 5:**
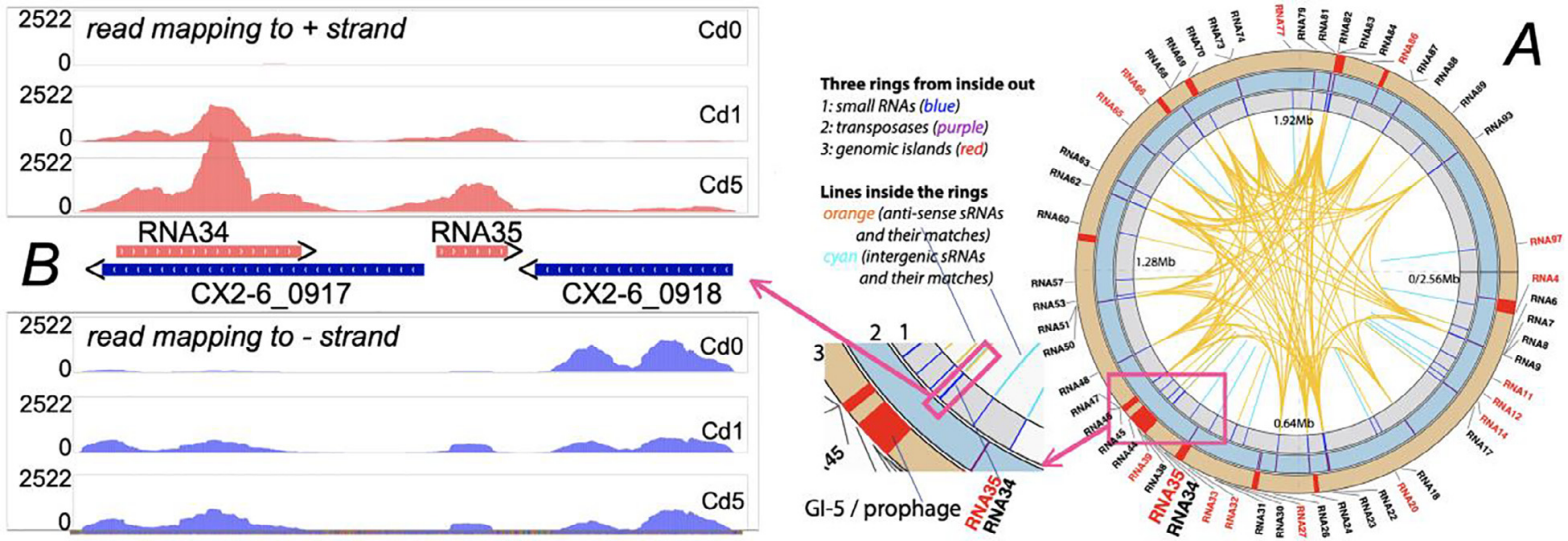
Noncoding RNAs in the main chromosome of CX 2–6. (A): The 55 ncRNAs contain 15 intergenic RNAs (igRNAs, red font) and 40 anti-sense RNAs (asRNAs, black font). The three rings are explained in the legend. The orange and cyan arch lines are also explained in the legend. All igRNAs only have one match in the genome, which is itself. Most asRNAs have multiple identical or near-identical matches in the genome. The area in the red frame is enlarged, which shows the location of the prophage that contains four RNAs (RNA34, 35, 38, 39). The area containing RNA34 and RNA35 is further enlarged and shown in B. (B): The enlarged view of the area that contains RNA34 (657 bp) and RNA35 (254 bp). RNA34 is an asRNA transcribed from the opposite strand of protein coding gene CX2-6_0917 (site-specific integrase), which is the last gene (3′ end) in the prophage. RNA35 is an igRNA located between CX2-6_0917 and CX2-6_0918 (also site-specific integrase), on the opposite strand of the two surrounding protein coding genes. The read mapping in the area is shown, with the positive strand (ncRNA genes) mapping shown on the top, and the negative strand (protein coding genes) mapping shown at the bottom. All the three conditions are shown as different panels with the read count being the y axis. (For interpretation of the references to color in this figure legend, the reader is referred to the web version of this article.)

In total 55 ncRNAs were identified (Fig. 5A), including 15 igRNAs and 40 asRNAs. These 55 ncRNAs contain 47 small ncRNAs (length <500 bp) and 8 longer ncRNAs (the longest is 1168 bp). Interestingly, 49 out of the 55 ncRNAs are differentially expressed genes (DEGs) in at least one condition pairs (e.g., Cd1-vs-Cd0, FDR < 0.05 and |log2FC| > 1) (Table S7). Only four igRNAs (RNA14, 27, 32, 77) and two asRNAs (RNA82, 89) are not DEGs. Most of ncRNA DEGs (44/49) are up-regulated under the cadmium stress. Fig. 5B shows the location of two example ncRNAs (RNA34 and 35) that were significantly up-regulated under cadmium stress. Both RNA34 and 35 are located at the end of the prophage (GI-5) and on the opposite strand of two site-specific integrases (*CX2-6_0917* and *CX2-6_0918*). RNA34 and RNA35 have almost no expression under Cd0, while CX2-6_0917 has very low expression under Cd0. However, RNA34 is the most highly expressed under Cd5, and most differentially expressed (Cd0-vs-Cd5 log2FC = 6.1) among the four genes in this region, followed by RNA35 (Cd0-vs-Cd5 log2FC = 6.0), and *CX2-6_0917* (Cd0-vs-Cd5 log2FC = 5.2), while *CX2-6_0918* is not a DEG (Table S7).

A BLASTN search to map the 55 ncRNAs onto the genome found that 27 (all up-regulated DEGs) out of the 40 asRNAs (38 DEGs) are mapped to the opposite strand of 18 transposase genes (CX2-6_0166, CX2-6_0545, CX2-6_0547, CX2-6_0586, CX2-6_0652, CX2-6_1003, CX2-6_1080, CX2-6_1194, CX2-6_1428, CX2-6_1456, CX2-6_1650, CX2-6_1694, CX2-6_1768, CX2-6_1932, CX2-6_1963, CX2-6_1973, CX2-6_2113, CX2-6_2259) (Fig. 5A and Table S7). These 18 transposases are mainly from two protein families (IS30 and IS110), and 7 of the transposases are DEGs in Cd0-vs-Cd1 comparison (FDR < 0.05 and |log2FC| > 1). One transposase (CX2-6_1003) is significantly down-regulated, while the other 6 are significantly up-regulated. These 18 transposases contain regions that are very similar to each other probably due to recent insertion events. Therefore, it is not surprising that each of the 27 asRNAs has 9 identical or near identical matches in different subsets of the 18 transposases (Fig. 5A and Table S7), aside from their own genomic locations.

All these findings suggest that ***E. faecium*** asRNAs play a significant role in cadmium stress response by modulating the expression of transposases. Additionally, 16 of the 27 asRNAs also have identical or near identical matches in the plasmid (Table S8) and all the matches are from transposases as well. Lastly, 4 ncRNAs (2 asRNAs and 2 igRNAs) are located in prophage (GI-5, Fig. 5A).

## 4 Discussion

The goal of this study was to use pan-genome analysis and comparative transcriptome analysis to understand the metabolic reprogramming of a newly isolated heavy-metal resistant stain CX 2–6 of *Enterococcus faecium*. Heavy metal resistance determinants have been well studied in various bacterial organisms (e.g., *Listeria monocytogenes* [19], [46], [47], [48]). *E. faecium* has been intensively studied as some of its strains are vancomycin-resistant and can cause serious infections in the human gastrointestinal tract, urinary tract, and bloodstream [49], [50], [51], [52]. However, other strains of *E. faecium* are beneficial probiotics and have been used in fermented dairy food production [53], [54], [55], [56]. A recent study has found that this species has an open pan-genome with different strains containing numerous environment-specific genes [57], due to their adaptions to various ecological environments.

In Fig. 4, the 138-genome phylogeny from our pan-genome analysis has revealed four major clades of *E. faecium* species. Inspecting the isolation source of the 138 (fully assembled) genomes found that most of the genomes in the red, green and blue clades were isolated from human blood, rectal swab, urine, feces of hospital patients (Table S1). In contrast, the pink clade, especially the sub-clade containing our CX 2–6 genome, consists of mostly non-pathogens from gut commensals and fermented foods (Fig. 4B). This agrees with the previous finding [57] that the dairy isolates and pathogenic *E. faecium* genomes are phylogenetically separated, even although our phylogeny was based on gene presence and absence, while their phylogeny was based on core gene alignment [57]. As CX 2–6 (soil isolated) is clustered with a group of strains from healthy human faces and fermented foods in East Asia, the CX 2–6 strain might have originated from food production and through human consumption of the food products entered the human faces and then farm soils. A susceptibility test on CX 2–6 found that it is susceptible to common alcohol-based disinfectants and antibiotics (Data S1). However, it is unclear if CX 2–6 is safe to be used as a potential probiotic in food production.

The most interesting findings from our comparative transcriptome analysis include the prophage highly activated by the Cd stress. Prophage activation as a response to various environmental stress has been extensively reported before (e.g., [58], [59], [60]). Our genome analysis revealed that a predicted 53-gene prophage (CX2-6_917-CX2-6_969) exists in the CX 2–6 genome (Fig. 3). The prophage may be longer than what PHASTER [30] predicted, as we observed that two surrounding genes CX2-6_916 and CX2-6_987 were annotated as site-specific recombinase/integrase. However, its exact boundary needs future experimental validation. Under the Cd stress, all but one of the 53 genes in the prophage are up-regulated DEGs (Table S3). More importantly, many of the most highly expressed genes of the genome (Fig. 3, ring 6) encode phage terminase for DNA packaging, capsid proteins, and head–tail connectors and adaptors (Table S3). This suggests that the prophage is actively transcribing to make virions (i.e., in the lytic phase). Interestingly, this prophage seems to be unique for the CX 2–6 genome (Fig. 4), as the orthologs of most genes in this prophage were absent in all other 137 fully assembled genomes. Searching this prophage among ∼2000 draft *E. faecium* genomes of RefSeq database found that it is only present in one genome (NCBI assembly ID GCF_002141355.1, and strain name 6H2_DIV0141), using a threshold that >70% of the query prophage’s proteins have homologs and are clustered in the subject genome. However, it is possible that this prophage and its mutated relics are present in more *E. faecium* genomes but exist in a very different form (e.g., some genes are lost, and others are dispersed in the host genomes). Given that prophage excision and movement can facilitate horizontal gene transfer and be a driver of rapid gain or loss of genetic material, it is possible that heavy metal exposure could enhance horizontal gene transfer by inducing prophage excision and movement.

Another important finding is that the Cd tolerance is a very conserved trait of *E. faecium*. The reason is that 75.4% (869/1152) DEGs responding to Cd treatment were found in the core genome of ***E. faecium*** (Fig. 4C). Previously, Cd resistance genes have been identified using various multi-omics data analysis [12], [13], [14], [15] in gram-positive bacteria. Our search of 121 previously published Cd resistance genes from two *Lactobacillales* species found that 78.5% of these published genes have 218 CX 2–6 homologs and 79.8% of these homologs are also found in all the 137 ***E. faecium*** genomes analyzed in our pan-genome analysis. In addition to the 218 homologs, our ultra-deep RNA sequencing (depth: 799–931X) revealed that almost half of the protein coding genes in CX 2–6 responded to the Cd stress. Some of the most well-known genes such as *adA* (CX2-6_0869) and other heavy metal translocating P-type ATPases (e.g., *zosA* [CX2-6_1471] and *ziaA* [CX2-6_1735]) in CX 2–6 are all highly up-regulated.

In fact, the top highly up-regulated DEGs (Fig. 2F) in Cd0-vs-Cd5 comparison are all transporters with *zosA* (CX2-6_1471) being the most highly up-regulated (adjusted P-value = 5.39E-119, log2FC = 6.49 and very high FPKM = 2770 under Cd5). This agrees with previous studies in other Cd resistant bacteria [12], [13], [14], [15] that transporters especially the P-type ATPase transporters are first responders to pump Cd to outside of the cell to prevent cell damages. In addition to these cation transporters, our GO enrichment analysis of up-regulated DEGs in Cd0-vs-Cd5 comparison (Fig. 2D) found that carbohydrate transporters and anion transporters are also most significantly up-regulated. This may indicate that these transporters are second responders to sequester and neutralize Cd in the outside of the cell, as pumping Cd to outside of the cell is not sufficient to keep the cell safe give higher concentration of Cd in the surrounding environment. Fig. 2C shows that the ribosome assembly-related functions are highly enriched in DEGs already up-regulated under Cd1 concentration. This is probably a response to the need to quickly make a large number of transporters and deploy them on the plasma membrane. As expected, the down-regulated DEGs are mostly related to nucleotide metabolism, which is in line with the fact that cellular growth is significantly slower under Cd stress (Fig. 1). Clearly, the CX 2–6 cells can rapidly reprogram their metabolisms (decrease growth but increase transporter production) to adapt to the presence of Cd in the environment.

Lastly, the role of ncRNAs in heavy metal resistance is very little studied, although small ncRNAs are known to be involved in stress response [61]. Riboswitches are considered as one special type of small ncRNA, as they are found in the 5′ end of mRNAs as the untranslated region (UTR) with *cis*-regulatory function to its mRNA [62]. Metalloregulatory riboswitches can bind to metals to regulate the expression of mRNAs [63]. However, in this study we focused on anti-sense RNAs (asRNAs) and intergenic RNAs (igRNAs). The RNA-seq read mapping-based approach has revealed 49 differentially expressed ncRNAs under the Cd stress. Interestingly, 27 up-regulated asRNAs are all located on the opposite strand of transposases. Although the exact function of these ncRNAs require further experimental study, the fact that these ncRNAs are significantly differentially expressed under Cd stress is intriguing. Our case study of RNA34 and RNA35, which are both located in the gene neighborhood of the prophage, indicates that these ncRNAs are likely involved in the prophage activation responding to the Cd stress.

## Author statement

Xin Cheng conceived and designed this study, performed all wet-lab experiments, drafted the manuscript, and secured funding. Bowen Yang performed all bioinformatics data analysis, created all figures and tables, and drafted the manuscript. Jinfang Zheng participated in the bioinformatics data analysis. Hongyu Wei participated in wet-lab experiments. Xuehuan Feng was involved in writing the manuscript. Yanbin Yin conceived and designed this study, participated in the bioinformatics data analysis, wrote the manuscript, and secured funding.

## Declaration of Competing Interest

The authors declare that they have no known competing financial interests or personal relationships that could have appeared to influence the work reported in this paper.
