# Supplementary figures and images for: Cadmium stress triggers significant metabolic reprogramming in Enterococcus faecium CX 2–6
Source: Comput Struct Biotechnol J. 2021 Oct 18;19:5678–87. doi: 10.1016/j.csbj.2021.10.021 (PMC8554106; doi:10.1016/j.csbj.2021.10.021)

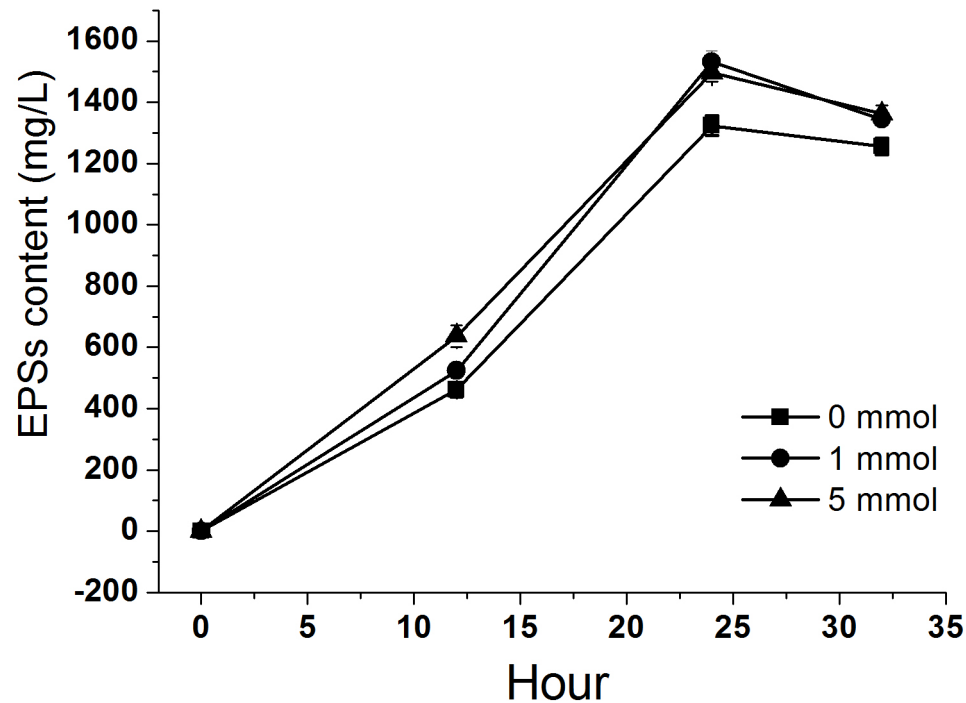

Supplement: Supplementary data 9 [file mmc9.pdf]
