# Supplementary material for: Cadmium stress triggers significant metabolic reprogramming in Enterococcus faecium CX 2–6
Source: Comput Struct Biotechnol J. 2021 Oct 18;19:5678–87. doi: 10.1016/j.csbj.2021.10.021 (PMC8554106; doi:10.1016/j.csbj.2021.10.021)
Supplement: Supplementary data 10 [file mmc10.pdf]

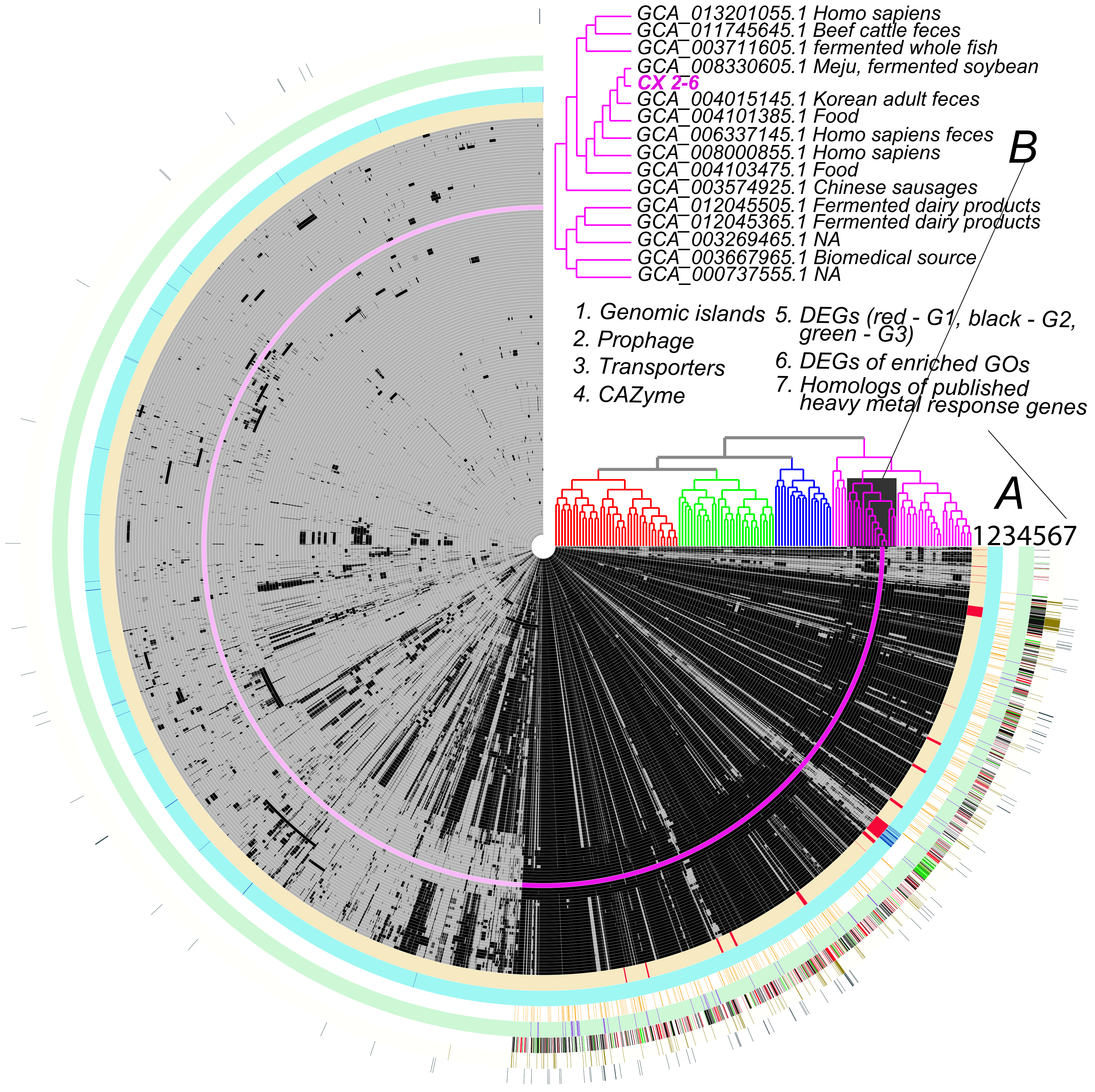

GCA_013201055.1 Homo sapiens
GCA_011745645.1 Beef cattle feces
GCA_003711605.1 fermented whole fish
GCA_008330605.1 Meju, fermented soybean
CX 2-6
GCA_004015145.1 Korean adult feces
GCA_004101385.1 Food
GCA_006337145.1 Homo sapiens feces
GCA_008000855.1 Homo sapiens
GCA_004103475.1 Food
GCA_003574925.1 Chinese sausages
GCA_012045505.1 Fermented dairy products
GCA_012045365.1 Fermented dairy products
GCA_003269465.1 NA
GCA_003667965.1 Biomedical source
GCA_000737555.1 NA
B
1. Genomic islands
2. Prophage
3. Transporters
4. CAZyme
5. DEGs (red - G1, black - G2, green - G3)
6. DEGs of enriched GOs
7. Homologs of published heavy metal response genes
A
1234567
