# Supplementary material for: Cadmium stress triggers significant metabolic reprogramming in Enterococcus faecium CX 2–6
Source: Comput Struct Biotechnol J. 2021 Oct 18;19:5678–87. doi: 10.1016/j.csbj.2021.10.021 (PMC8554106; doi:10.1016/j.csbj.2021.10.021)
Supplement: Supplementary data 11 [file mmc11.doc]

**Data S1: Susceptibility tests of CX 2-6**

**Alcohol-based disinfectants**

The three most common alcohol-based disinfectants, ethanol, isopropanol, and *n*-propanol [1], were used and placed into three groups [2]: (i) group A: ethanol, (ii) group B: ethanol + isopropanol (ethanol: isopropanol = 6:1 (v/v)), and (iii) group C: ethanol + *n*-propanol (ethanol: *n*-propanol = 6:1 (v/v)). Briefly, the solution of each group was diluted to 85%, 87.5%, 90%, 92.5%, 95%, 97.5%, 100%. Then 2.5 ml or 3 ml dilutions of each group were fixed to 5 ml by MRS medium of different concentrations. At the end, the concentration of alcohol-based disinfectants in test tube 1-10 was 46.25%, 47.5%, 48.25%, 50%, 51%, 52.5%, 54%, 55.5%, 57%, 58.5%, 60%. Finally, 0.1 mL of bacterial suspension containing approximately 1×10^8^ CFU/mL was transferred into each test tube. All test tubes were then incubated at 37℃ for 24h and the absorbance at 600 nm of bacterial solution was used to indicate the activity of alcohol-based disinfectants.

The figure below shows that CX 2-6 is susceptible to alcohol-based disinfectants particularly when ethanol is combined with isopropanol or *n*-propanol. The disinfectant concentration at ~55% and higher is recommended.


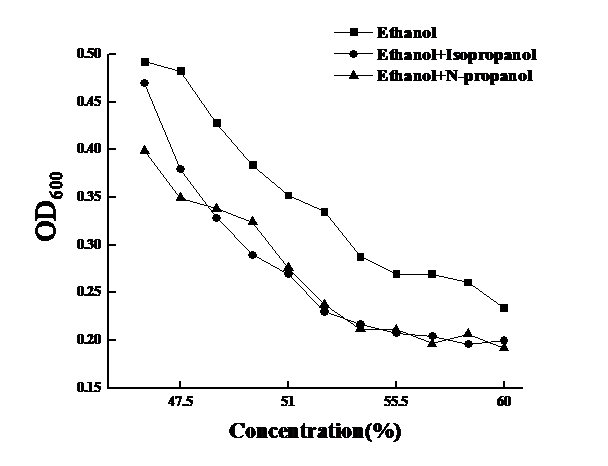


**Antibiotics**

According to [3], a total of 12 antibiotics were used for MIC (Minimum Inhibitory Concentration) measurement, following the broth microdilution recommended by the Clinical and Laboratory Standards Institute (CLSI) [4,5]. The Table below shows that CX 2-6 is susceptible to the treatment of all the 12 antibiotics except for Erythromycin.

| Antibiotics | MIC_50_/（mg·L^-1^） | MIC_90_/（mg·L^-1^） |
| --- | --- | --- |
| Penicillin | ≥0.125 | ＞1 |
| Ampicillin | ＞0.25 | ≥2 |
| Chloramphenicol | ＞0.25 | ≥32 |
| Tetracycline | ＞0.25 | ≥64 |
| Erythromycin | —— | —— |
| Streptomycin | ≥128 | —— |
| Kanamycin | ≥16 | ＞128 |
| Vancomycin | ＞0.125 | ≥32 |
| Ciprofloxacin | ＞4 | —— |
| Nitrofurantoin | ＞1 | —— |
| Lincomycin | ＞4 | ＞16 |
| Rifampicin | ＜0.125 | ＞0.25 |

Note: In the table, "——" indicates poor inhibition or no significant difference between treatments.

References

[1] McDonnell G, Russell A D. Antiseptics and disinfectants: activity, action, and resistance[J]. Clinical microbiology reviews, 1999, 12(1): 147-179.

[2] SU Yu-xin，WEI Qiu-hua，REN ZheDetection of drug-resistance gene of MRSA and *Acinetobacter baumannii* and their resistance to three kinds of alcohol disinfectants(in chinese) [J]. Military Medical Sciences, 2012, 36(11): 851-861.

[3] Beier R C, Duke S E, Ziprin R L, et al. Antibiotic and disinfectant susceptibility profiles of vancomycin-resistant *Enterococcus faecium* (VRE) isolated from community wastewater in Texas[J]. Bulletin of environmental contamination and toxicology, 2008, 80(3): 188-194.

[4] Clinical and Laboratory Standards Institute. Performance standards for antimicrobial disk and diffusion susceptibility tests for bacteria isolated from animals:second informational supplement VET01-S2[R]. Wayne:CLSI, 2013．

[5] Clinical and Laboratory Standards Institute. Performance standards for antimicrobial susceptibilit- y testing: twenty-third informational supplement M100-S23[R]. Wayne:CLSI, 2013．
